# 

**Published:** 1845-01

**Authors:** 


### BRITISH AND FOREIGN MEDICAL REVIEW

OR

### QUARTERLY JOURNAL

OF

### PRACTICAL MEDICINE AND SURGERY

STATE OF LOUISIANA CHARITY HOSPITAL

JUSTICE

MEDICAL LIBRARY.

LIBRARY OF THE

Orleans Parish Medical Society

EDITED BY

JOHN FORBES M.D. F.R.S. F.G.S.

---

VOL. XIX

JANUARY—APRIL 1845

LONDON

JOHN CHURCHILL PRINCES STREET SOHO

MDCCCXLV.

C. AND J. ADLARD, PRINTERS, BARTHOLOMEW CLOSE.

### BOOKS RECEIVED FOR REVIEW.

1. Introduction to the Study and Practice of Midwifery. By William Campbell, M.D. and Alexander D. Campbell, B.A. Second Edition.—Edinburgh, 1843. 8vo, pp. 809.

2. Observations on the Treatment of some forms of Incipient Phthisis. By Joseph Bell, Surgeon, Glasgow.—Glasgow, 1844. 8vo, pp. 22.

3. Mémoires pour servir a l'étude des maladies des Ovaires. Premier Mémoire. Par Achille Chereau, M.D.—Paris, 1844. 8vo, pp. 189.

4. First Report of the Commissioners for inquiring into the state of Large Towns. Two vols. 8vo.—London, 1844.

5. Rapport sur l'Organisation de la Médecine en Allemagne. Par M. le Dr. Roger. Juin, 1842. —Paris.

6. Supplementary Report on the practice of Interment in Large Towns. By E. Chadwick.—London, 1843. 8vo.

7. The Educational provisions of the Birmingham School of Medicine. By the Rev. V. Thomas, B.D.—Oxford, 1843. 8vo. 1s.

8. Researches into the Physical History of Mankind. By J. C. Prichard, M.D. F.R.S., &c. Third Ed. Vol. IV.—Lond. 1844. 8vo, pp. 631. 18s.

9. Lœrebog i Fædselsvidenskaben for Jordenærdre. Ved Dr. F. C. Faye.—Skien, 1844. 8vo, pp. 241.

10. Handbuch der Anatomischen Chirurgie. Von W. Rozer.—Tübingen, 1844. 8vo, pp. 588.

11. Traité Pratique d'Auscultation. Par MM. Barth et Royer. Séconde Edition.—Paris, 1844. 8vo, pp. 686.

12. Vestiges of the Natural History of Creation. —London, 1844. 8vo, pp. 390. 7s. 6d.

13. Contributions to Ophthalmic Surgery. Part II. By W. R. Wilde, M.R.I.A.—Dublin, 1844. 8vo, pp. 38.

14. Contributions to Aural Surgery. By W. R. Wilde, M.R.I.A.—Dublin, 1844. 8vo, pp. 35.

15. Lehrbuch der Speciellen Nozologie und Therapie. Von C. H. Fuchs, Professor zu Göttingen. Erste Lieferung.—Göttingen, 1844. 8vo, pp. 160.

16. The Principles of Surgery. By James Miller, F.R.S.E. Professor of Surgery in the University of Edinburgh.—Edinburgh, 1841. 8vo, pp. 716. 9s.

17. Memoir on the Sex of the Child as a cause of difficulty and danger in Human Parturition. By James Y. Simpson, M.D. F.R.S., &c.—Edin. 1844. 8vo, pp. 55. (From the Ed. Journ.)

18. Percussion und Auscultation des Herzens im gesunden und kranken Zustande, &c. Von L. Günzburg.—Wien, 1844. 8vo, pp. 179.

19. A Manual of Elementary Chemistry, Theoretical and Practical. By George Fownes, with numerous wood engravings.—London, 1844. 8vo, pp. 566. 12s. 6d.

20. A Practical Treatise on Midwifery; exhibiting the present advanced state of the science. By F. J. Moreau. Translated from the French. by T. F. Betton, M.D. and P. B. Goddard, M.D. With 80 Plates.—Philadelphia, 1844. 4to, pp. 235. 10 dollars.

21. Principles of Forensic Medicine. By W. A. Guy, M.B. &c. Part III. 2s. 6d.

22. Remarks on the Report of the Commissioners on the Poor Laws of Scotland. By W. P. Alison, M.D. &c.—Edinb. 1844. 8vo, pp. 302.

23. Facts and Observations in Medicine and Surgery. By John Grantham, Surgeon.—Lond. (no date) 1844? 8vo, pp. 216. 7s. 6d.

24. Principles of Human Physiology. By W. B. Carpenter, M.D. F.R.S., &c. Second Edition.—London, 1844. 8vo, pp. 745. 20s.

25. Outlines of Military Surgery. By Sir G. Ballinghall, M.D., &c. Third Edition.—Edinb 1844. 8vo, pp. 568. 14s.

26. A Dictionary of Terms used in Medicine and the Collateral Sciences. By R. D. Hoblyn, A.M. Second Edition, revised and enlarged.—London, 1844. 8vo, pp. 394. 10s.

27. Remarks upon the Mortality of Exeter; together with suggestions towards the improvement of the Public Health. By F. Shapter, M.D. —London, 1844. 8vo, pp. 32. 1s.

28. On Military Hygiene, and particularly upon the Clothing of Soldiers. By Frederic Roberts, esq. Assistant-Surgeon 59th Regiment.—Portsmouth, 1844. 8vo, pp. 24.

29. The Medical Remembrancer, or Book of Emergencies. Second Edition.—London, 1845. 18mo, pp. 108. 2s. 6d.

30. Practical Observations and Suggestions in Medicine. By Marshall Hall, M.D. F.R.S.—Lond. 1845. 8vo, pp. 360. 8s. 6d.

31. Text Book of Anatomy for Students. By A. T. Lizars, M.D. Part III.—Edinburgh, 1844.

32. A Lecture on the Ethnology of the Ancient Irish. By W. R. Wilde.—Dublin, 1844. 8vo, pp. 16.

33. A Report on the Obstetric practice of University College Hospital, London. By E. W. Murphy, M.D., Professor of Midwifery, &c.—Dublin, 1844. 8vo, pp. 53.

34. An Address to the Harveian Society on the advantages of Medical Association. By E. W. Murphy, M.D., &c.—London, 1844. 8vo, pp. 15.

35. Londres et les Angles des Temps Modernes. Par le Docteur Bureaud Rioffrey.—London, 1844. 8vo, pp. 430.

36. A Practical Treatise on the Diseases peculiar to Women. By J. Ashwell, M.D.—London, 1844. 8vo, pp. 737. 4s.

37. Pathologia Indica, or the Anatomy of Indian Diseases. Part I. By Allen Webb, B.M.S. Lecturer on Surgery in the Calcutta College of Medicine.—Calcutta, 1844. 8vo, pp. 204. 1s.

38. Ricerche ed esperimenti intorno alla formazione della cotenna nel sangue, &c. Del Dottor G. Polli.—Milano, 1843. 8vo, pp. 167.

39. Lectures on Pulmonary Phthisis. By J. T. Evans, M.D.—Dublin, 1844. 8vo, pp. 196.

40. Urinary Deposits, their Diagnosis, Pathology, and Therapeutical Indications. By Golding Bird, A.M. M.D., &c.—Lond. 1844. 8vo, pp. 323. 8s.

41. An Apology for the Nerves; or their Influence and Importance in Health and Disease. By Sir George Lefevre, M.D., &c.—London, 1844. 8vo, pp. 363. 9s.

42. Causes Générales des Maladies Chroniques, Specielment de la Phthisie Pulmonaire, &c. Par A. Fonscault, &c.—Paris, 1844. 8vo, pp. 480.

